# Convergent molecular pathways and shared genetic mechanisms in classic syndromic autism spectrum disorders: a narrative review

**DOI:** 10.3389/fgene.2026.1858001

**Published:** 2026-10-30

**Authors:** Ryan Bourgaize, Aderonke Oyetunji, Merlin G. Butler

**Affiliations:** 1 College of Osteopathic Medicine, Kansas City University, Kansas City, MO, United States; 2 Department of Psychiatry and Behavioral Sciences, University of Kansas Medical Center, Kansas City, KS, United States

**Keywords:** neurodevelopmental/behavioral/psychiatric disorders, overlapping genes, protein-protein interactions and processes, shared genetic mechanisms and protein networks, STRING and BioGRID web-based programs and databases, syndromic autism, therapeutic interventions

## Abstract

Autism spectrum disorders (ASD) are defined as a group of neurodevelopmental disorders that share common core features including restrictive repetitive stereotypic behavioral patterns and interests affecting social communication. Over 1,200 genes have been associated with ASD and impacted by copy number variants, chromosomal, and/or single gene defects or epigenetics. Genetic factors contribute to most cases of autism. At least twelve classic genetic disorders with autism as a common feature were selected involving 31 causative or implicated genes with 30 recognized as ASD genes. These disorders were selected to represent a range of genetic abnormalities such as chromosomal deletions, duplications, or trisomy, as well as single or multiple defective genes often with inter-relationship to other genes. Collectively, these syndromes present with neurodevelopmental/behavior/psychiatric findings with ASD, ADHD, intellectual disability and/or psychosis as features. This narrative review is novel by synthesizing available evidence on shared genes, molecular pathways, and protein-protein interaction networks across twelve classic syndromic autism spectrum disorders. Drawing on existing literature and curated web-based databases including STRING and BioGRID, nineteen of the 31 causative and/or implicated genes identified across syndromic autism disorders showed overlap across the twelve classic syndromes having different genetic defects and converged on shared molecular functions, pathways and biological processes. These related to chromatin remodeling, RNA regulation of neurodevelopment and function, synaptic signaling, neuronal connectivity, and protein homeostasis. Phelan-McDermid, Fragile X, Rett, Prader-Willi, Angelman, Burnside-Butler, and Cornelia de Lange syndromes exhibited overlapping pathways related to typical features of autism including social behavior, language acquisition, and associated neurodevelopment. Novel observations of shared molecular patterns across classic genetically distinct syndromes with ASD showed disturbances suggestive of unified, pathway-based therapeutics requiring more investigations to inform future treatment strategies.

## Introduction

Autism spectrum disorders (ASD) represent a clinically varied group of neurodevelopmental conditions that share common behavioral core features, including restrictive and repetitive stereotypic behavioral patterns and interests affecting social communication (Sauer et al., 2021). A growing number of individuals with ASD are now diagnosed with genetic defects, dysmorphism, or syndromic features with or without chromosomal abnormalities using advanced genomic testing (Genovese and Butler, 2020).

ASD can be broadly categorized into idiopathic and syndromic forms (Genovese and Butler, 2023). Risk factors included genetic and non-genetic modulators (Rylaarsdam and Guemez-Gamboa, 2019; Miles, 2011; Jones et al., 2013) and genetic factors that contribute significantly to developing ASD such as copy number variants (CNV), chromosomal defects, and single or multiple gene variants or alterations (Genovese and Butler, 2023). Rett and Down syndromes and cytogenetic deletion disorders are related to ASD because each involves pathogenic variants or chromosomal regions that may contain more than one gene with disruption of their encoded proteins ostensibly impacting neurodevelopmental pathways and functions underlying autism.

The clinical presentation of autism within classic genetic syndromes can be found in chromosomal anomalies or rearrangements cytogenetic including deletions (e.g., Prader-Willi, Williams, DiGeorge, Cornelia de Lange, Phelan-McDermid), duplications (e.g., 15q11-q13) or trisomy 21 (Down syndrome) (Miles, 2011; Jones et al., 2013; Woodbury-Smith et al., 2018; Ho et al., 2016; Butler et al., 2015; Fernandez and Scherer, 2017; Tammimies et al., 2015; Sztainberg et al., 2016; Herman et al., 2007; Genovese and Butler, 2024; Hodges et al., 2020; Forrest and Penzes, 2020). Chromosomal regions associated with autism, but not limited are: 1p36.22, 1q24.2, 2p13.1, 2q13, 2q37.3, 3p26.2, 4q34.2, 6q24.3, 6q27, 7q35, 8q24.22, 9p21.3, 9q31.2, 12p13.31, 13q13.2-q22, 15q11, 15q11-q13, 15q22, 16p11.2, 16p13.2, 17p11.2, 18q21.1, 22q11, and Xp22. High-resolution chromosomal microarrays and genomic technology have led to identifying additional chromosomal regions, including 15q11.2 BP1-BP2 deletions (Burnside-Butler) and duplications. Gene defects in autism include single autosomal recessive (e.g., Phenylketonuria [PKU]), autosomal dominant (e.g., Tuberous Sclerosis), imprinting errors (e.g., Angelman), triplet repeat expansion (e.g., Fragile X) and chromosome abnormalities (e.g., Down) (Sauer et al., 2021; Hodges et al., 2020).

A literature review in 2015 reported approximately 800 genes associated with autism and their locations assigned to high-resolution chromosome ideograms (Butler et al., 2015) and used throughout. More recently, the SFARI (Simons Foundation Autism Research Initiative) (https://www.sfari.org), and SFARI database (https://gene.sfari.org) cataloged research genes implicated in autism susceptibility and lists over 1,200 genes. For example, of these genes and genetic syndromes associated with ASD, over 100 autism-risk genes were used in clinical genetic laboratory testing (Jones et al., 2013; Woodbury-Smith et al., 2018; Ho et al., 2016; Forrest and Penzes, 2020). Of the twelve syndromic ASD conditions selected for study with different classes of genetic defects, 31 genes were found to be either causative or implicated as noted in several sources (e.g., www.omim.org; www.GeneReviews.org; www.GeneCards.org; www.SFARI.org) and references (Sauer et al., 2021; Genovese and Butler, 2020; Genovese and Butler, 2023; Rylaarsdam and Guemez-Gamboa, 2019; Miles, 2011; Jones et al., 2013; Butler et al., 2015; Forrest and Penzes, 2020).

Syndromic autism refers to those with ASD diagnosed in the context of clinical and behavioral features and a genetically confirmed syndrome with ASD as a common finding. Each disorder has a clinically defined pattern of somatic abnormalities along with a neurobehavioral phenotype often including ASD (Genovese and Butler, 2020; Fernandez and Scherer, 2017). Examples of syndromic disorders with ASD chosen for our study included Fragile X, Tuberous Sclerosis, Phelan-McDermid, Prader-Willi, Angelman, DiGeorge, Phenylketonuria, Rett, Down, Williams, Burnside-Butler and Cornelia de Lange syndromes (Jones et al., 2013; Fernandez and Scherer, 2017; Sztainberg et al., 2016; Genovese and Butler, 2024; Morimoto et al., 2021; Butler and Duis, 2020; Rafi and Butler, 2020; Godler et al., 2025; Butler, 2023; Genovese A. C. and Butler M. G., 2025; Butler, 2026; Kline et al., 2018; Lyst and Bird, 2015; Schenck et al., 2001; Kolevzon et al., 2014; Blau et al., 2010; Coffee et al., 2009; Sarasua et al., 2011; Butler and Meaney, 2005; Halfon and Kuo, 2013; Ousley et al., 2017; Abbeduto et al., 2019; Nakanishi et al., 2012; Toma et al., 2014; Genovese A. and Butler M. G., 2025; Zoghbi and Bear, 2012; Darnell et al., 2011). Attempts to differentiate syndromic ASD into clinical and molecular categories included further classification into autistic or behavioral phenotypes (Tammimies et al., 2015; Sztainberg et al., 2016). However, there is a limited understanding of the role of most ASD-associated genes and their interactions, molecular mechanisms, biological processes and pathways in common with molecular or genetic characterization, stimulating the focus of our study.

Genetic factors can influence the activity and function of other genes including their encoded proteins but presumably can share common pathways with regulatory mechanisms that impact expression through use of transcription factors, methylation or histone modifications. Genes that play a role in syndromic ASD may also influence other genes through intergenic interactions and potentially involve complex gene or protein networks where expression of one or more genes can affect the activity of regulatory networks, shared mechanisms or pathways and their related functions (Genovese and Butler, 2023; Rylaarsdam and Guemez-Gamboa, 2019; Genovese and Butler, 2024; Toma et al., 2014).

ASD affects at least 1% of children in the United States population, with a 4:1 male-to-female ratio. About one-fourth of children with autism are diagnosed by two to 3 years of age, and 60% show intellectual disabilities. Genetics play a significant role in the causation of ASD and estimated as high as 90 percent (Herman et al., 2007). Hence, the importance of investigating genetics in ASD is emphasized with expanded use of advanced genetic testing with next-generation DNA sequencing of disease-specific disorders or with whole exome and high-resolution microarrays to better understand the etiology of ASD (Ho et al., 2016). The description of ASD was characterized according to DSM-5 diagnostic criteria with deficits in social communication and restricted, repetitive behaviors (Halfon and Kuo, 2013).

We concentrated on syndromic ASD arising from chromosomal anomalies (e.g., trisomy), microdeletions/duplications, and single gene defects which impact neurodevelopment, cognition, and behavior. The prevalence of ASD varied across syndromic ASD, for example, the rates are estimated at 70%–80%, ∼30%, ∼30%, up to 50%, >50%, and ∼40%, respectively for Phelan-McDermid, Prader-Willi, Angelman, DiGeorge, Fragile X, and Cornelia de Lange syndromes (Jones et al., 2013; Godler et al., 2025; Kolevzon et al., 2014; Butler and Meaney, 2005; Ousley et al., 2017; Abbeduto et al., 2019; Nakanishi et al., 2012; Toma et al., 2014; Genovese A. and Butler M. G., 2025; Zoghbi and Bear, 2012; Darnell et al., 2011). Furthermore, these findings underscored the strong genetic influence underlying syndromic ASD with emphasizing how gene-disruptive events, encoded protein interactions and networks altered molecular functions that could shape behavioral and cognitive phenotypes. Although many genes are associated with ASD, several classic genetic syndromes with ASD as a clinical feature have been under studied. There remains a limited understanding of how ASD-associated genes and their encoded proteins overlap across clinically distinct syndromic conditions, specifically with a variety of different genetic defects. These include chromosomal deletions or duplications, imprinting errors, triplet repeat expansion, autosomal recessive or dominant transmission patterns, and single gene variants. By studying classic genetic syndromic ASD with recognized genetic defects, we reduce heterogeneity compared to idiopathic ASD. Ultimately, through our search, we planned to provide information and guide treatment for individuals with syndromic ASD by identifying autism-associated genes and shared genetic mechanisms, interactions, functions, and pathways (Genovese and Butler, 2024).

Network-based computational approaches are now available to examine functional relationships among disease-associated genes and identify convergent molecular mechanisms across genetically heterogenous conditions including autism. For example, autism and macrocephaly were recently analyzed and the role of *PTEN* gene characterized (Genovese A. and Butler M. G., 2025). The STRING and BioGRID web-based programs were selected in our study to search existing data and integrate curated experimental and predicted interactions. This enabled systematic analysis of molecular functions, biological processes, and pathways with statistical enrichment metrics and p values including false discovery rate corrections. Rather than cataloging individual gene annotations, our study focused on cross-syndrome integration to identify shared molecular networks and pathways of clinically distinct genetic disorders unified by autism as a common phenotype.

Table 1 summarizes twelve representative classic syndromic ASD conditions selected for review, including their genetic defects, causative or implicated genes, prominent clinical features, and reported ASD prevalence. This approach is unique and addresses a critical gap, particularly whether the diverse genetic lesions underlying these syndromic conditions converge on common molecular systems that may collectively explain the shared presence of ASD across the twelve syndromes. Identifying such convergence is not only scientifically meaningful but has direct clinical implications, as shared pathways represent potential targets for therapeutic interventions that may benefit individuals across multiple syndromic ASD conditions rather than limited to a single disorder. Understanding the molecular basis of ASD within the context of these well-defined syndromic conditions offers a powerful and novel lens through which to investigate the causation of both the syndrome-specific findings and ASD as a major shared phenotype with the ultimate goal of advancing research and informing the development of common therapeutic strategies targeting the behavioral and neurodevelopmental features shared across these distinct disorders.

**TABLE 1:** Clinical and genetic characteristics of twelve classic syndromic autism spectrum disorders.

Syndrome	Genetic defect	Causative/Implicated genes	Prominent clinical features	ASD prevalence
Fragile X	Triple CGG repeat expansion	FMR1	Intellectual disability, macro-orchidism, large ears and characteristic facies, connective tissue dysplasia, social anxiety	>50%
Rett	Single gene	MECP2	Regression of skills, hand stereotypies, seizures, loss of speech	∼60%
Phelan-McDermid	22q13 deletion	SHANK3	Absent/delayed speech, hypotonia, intellectual disability, dysplastic ears pointed chin, dolichocephaly with dysmorphism	∼80%
Prader-Willi	15q11-q13 paternal deletion/imprinting	SNRPN, MAGEL2	Infantile hypotonia and failure to thrive, hypogonadism/genitalism, childhood hyperphagia, short stature, small hands/feet, obesity, self injury, intellectual disability	∼30%
Angelman	15q11-q13 maternal deletion/imprinting	UBE3A	Seizures, ataxia, absence of speech, bouts of laughter, characteristic facies, intellectual disability	∼30%
DiGeorge	22q11 deletion	PRODH, COMT, HIRA, TBX1	Velo-cardio-facial anomalies, palatal defects, immune deficiency, learning difficulties	∼50%
Williams	7q11.23 deletion	GTF2I, GTF2IRD1, ELN, LIMK1	Hypersociability, seizures, intellectual disability, cardiovascular defects, distinctive craniofacial features with prominent nose and lips and hoarse voice	∼20%
Tuberous Sclerosis	Single gene	TSC1, TSC2	Benign tumors, seizures, hamartomatous skin nodules and phakomata with intellectual disability	∼50%
Down	Trisomy 21	DYRK1A, SOD1, APP, HMGN1	Intellectual disability, hypotonia, characteristic flat facies, slanted palpebral fissures, small ears and cardiac defects	∼20%
Phenylketonuria	Single gene	PAH	Intellectual disability, if untreated, fair complexion, seizures, behavioral problems	∼20%
Burnside-Butler	15q11.2 BP1-BP2 deletion	NIPA1, NIPA2, CYFIP1, TUBGCP5	Neurodevelopmental, motor and speech delay, behavioral problems, seizures, psychiatric features	∼30%
Cornelia de Lange	Several cohesin-related genes	SMC1A, SMC3, NIPBL, RAD21, HDAC8, ANKRD11	Growth retardation, shortened limb defects, characteristic facies with synophrys, down-turned upper lip, anteverted nares, intellectual disability	∼40%

Data compiled from established clinical and genetic literature sources including OMIM (www.omim.org), GeneReviews (www.genereviews.org), GeneCards (www.genecards.org), and Smiths Recognizable Patterns of Human Malformation (Jones et al., 2013).

Prevalence data are largely derived from studies conducted in Western or Caucasian populations. Comprehensive global and population specific prevalence data for many of these rare syndromic conditions remains limited in the published literature.

This narrative review draws on established literature and curated databases and web-based programs including STRING and BioGRID to synthesize evidence on shared genes, molecular mechanisms, and pathways across the classic syndromes with varied genetic defects in which autism is a well-documented clinical feature with the goal of identifying convergent biological themes that may inform future research and therapeutic development.

## Review of data strategy

### Identification and selection of syndromic ASD and their genes

To identify genes that are causative or implicated in classic syndromic autism spectrum disorders, a targeted literature-based review was performed drawing from multiple established clinical and genetic resources. Syndrome selection was guided by the requirement that autism or ASD is a well-documented feature of the condition, and that causative or strongly implicated genes are recognized in the literature (e.g., 5-16). Candidate syndromes and their associated genes were identified through keyword-based searches of PubMed (www.pubmed.org) using terms such as ‘syndromic autism’, ‘autism spectrum disorder’, and each individual syndrome name. Additional gene and syndrome information was drawn from the Online Mendelian Inheritance in Man database (OMIM; www.omim.org), GeneReviews (www.genereviews.org), GeneCards (www.genecards.org), and Smith’s Recognizable Patterns of Human Malformation (Jones et al., 2013). The SFARI gene database (https://gene.sfari.org), which catalogs over 1,200 genes implicated in ASD susceptibility, was also incorporated to further confirm ASD gene status for each identified gene.

Genes were classified as causative if pathogenic variants or chromosomal alterations were known to directly cause an associated genetic syndrome in which autism is a documented clinical feature (Genovese and Butler, 2020; Genovese and Butler, 2023; Jones et al., 2013; Fernandez and Scherer, 2017). Furthermore, genes were classified as implicated if evidence from literature supported a biological or functional role in the syndrome or ASD phenotype, but the gene was not considered sufficient to cause the disorder as other genes were also implicated. Both causative and implicated genes were included to capture the full spectrum of genetic contributors relevant to syndromic autism. Through this process, twelve classic syndromic ASD conditions were selected with clinical genetics input representing a broad range of genetic mechanisms including single-gene disorders, chromosomal deletions, copy number variants, imprinting errors, triple repeat expansions, and trisomy (e.g., 6,14,31). Thirty-one causative or implicated genes were identified across the twelve syndromes.

### Searchable web-based integrated genetic and protein mechanisms in syndromic ASD

To characterize the molecular functions, biological processes, pathways, protein-protein interactions, and disease associations of the identified genes, existing data from two complementary curated web-based platforms were reviewed and synthesized. These platforms were selected because of their ability to integrate curated experimental and predicted interaction data, enabling systematic analysis of molecular functions, biological processes, and pathways with established statistical enrichment metrics including p-values with false discovery rate correction.

The well-established STRING database (https://string-db.org) provides a tiered framework to analyze existing integrated protein-protein interaction networks derived from multiple evidence and sources including genomic context predictions, high-throughput experimental datasets, automated text mining of published literature, and conserved co-expression patterns (Szklarczyk et al., 2023; Szklarczyk et al., 2025). A confidence score threshold of 0.8 was applied in the STRING network analysis, representing high confidence protein-protein interactions on a scale of 0–1, to ensure that only the most reliably supported interactions were included in the network visualization. Each node in the STRING network represents all protein isoforms produced by a single gene, and each assigned edge reflects a meaningful predicted or known biological association, either physical or functional, derived from one or more evidence sources. For each of the 31 identified genes, existing STRING data were searched and reviewed at the first-tier level, which captures ten primary interacting protein nodes per gene. It then provides information on mechanisms, biological processes, molecular functions, cellular components, pathways, and disease-gene associations. Gene overlap across syndromic ASD conditions was defined as the presence of the same gene appearing in the first-tier interacting protein network consisting of ten related proteins involved in two or more of the selected syndromic conditions.

The STRING-derived information uses a step-by-step approach for statistical analysis. Initially, this includes a *Count in network* category (e.g., biological processes) and indicates how many proteins are in network and annotated with a particular term along with how many total proteins have this term assigned to this variable per category. Second, *Strength* is log10(observed/expected) measure which describes how large the enrichment effect might be based on the ratio between the number of proteins in the network that are annotated with a term and the number of proteins expected to be annotated with that term in a random network of the same size. Third, *Signal* is defined as a weighted harmonic mean between the observed/expected ratio and -log (FDR or false discovery rate). Fourth, *FDR* is a statistical measurement that examines the significance of enrichment shown as p-values corrected for multiple testing within each category. We searched each of the 31 causative or implicated genes (e.g., *MECP2* and Rett syndrome) for humans (*Homo sapiens*) only during the month of July 2025.

A second searchable web-based BioGRID (https://thebiogrid.org program was used as a public access database of archive curated data for functional protein-protein interactions. This searchable program identifies related proteins assembled from relevant sources and updated for analysis including Gene Oncology biological processes, Gene Ontology molecular functions and Gene Ontology cellular components. Five genes and their encoded protein were selected with each representing a distinct genetic defect and syndrome including *SHANK3* (Phelan-McDermid), UBE3A (Angelman), *TSC1* (Tuberous Sclerosis), *SOD1* (Down), and *LIMK1* (Williams). These genes and their encoded protein were examined using BioGRID and selected based on their established causal roles in classic syndromic autism along with their recognition as ASD-associated genes representing a distinct genetic defect and mechanism while converging on shared neurodevelopmental pathways. These five genes were selected as representative examples to illustrate the breadth of molecular convergence across the twelve syndromes rather than to provide an exhaustive BioGRID analysis of all 31 genes. Of the 31 genes examined, 30 are recognized as ASD related genes in established databases. PAH, the causative gene in Phenylketonuria, is the single gene among the 31 not currently recognized as an ASD gene.

Together, these two established web-based platforms provide a complementary and comprehensive basis for synthesizing existing evidence on molecular convergence across the twelve selected syndromic ASD conditions with shared neurodevelopmental pathways. This approach allowed for identification of shared gene networks, protein-protein interaction patterns, and convergent biological pathways from existing curated literature and database sources rather than the generation of novel experimental findings for our narrative review.

## Results and discussion

### Shared molecular mechanisms and pathways in syndromic autism

A synthesis of existing literature and curated database evidence from two separate established web-based computer programs (STRING and BioGRID) were used across twelve selected classic syndromic ASD conditions with a wide range of genetic defects used to search and examine existing data. Our search interestingly revealed that 30 of the 31 causative or implicated genes in the syndromes were recognized as ASD-related genes (www.sfari.org; www.omim.org; www.GeneReveiws.org; www.GeneCards.org) (Sauer et al., 2021; Genovese and Butler, 2020; Genovese and Butler, 2023; Rylaarsdam and Guemez-Gamboa, 2019; Miles, 2011; Jones et al., 2013; Woodbury-Smith et al., 2018; Ho et al., 2016; Butler et al., 2015; Forrest and Penzes, 2020; Morimoto et al., 2021; Butler and Duis, 2020; Rafi and Butler, 2020; Godler et al., 2025; Butler, 2023). Nineteen of these 31 genes showed overlap across two or more syndromic conditions with 15 of the 19 genes established as ASD genes. In summary, the 19 overlapping genes were *SNRPN, UBE3A, FMR1, FXR1, FXR2, CYFIP1, SHANK3, RAD21, SMC1A, SMC3, TSC2, AKT1, DDC, DLG1, DLG4, NIPA1, NIPA2, TUBGCP5* and *RAD23A*. They converged on shared molecular functions spanning chromatin remodeling, RNA regulation, synaptic signaling, and protein homeostasis, as detailed in the sections below. These findings underscored that despite the broad genetic heterogeneity across syndromic ASD conditions, a limited set of core molecular systems appeared to be recurrently disrupted, suggesting that shared pathophysiological mechanisms may underline the overlapping neurodevelopmental and behavioral phenotypes observed across these clinically distinct genetic disorders (Sonawane et al., 2021; Remeseiro and Losada, 2013; Ozçelik et al., 1992; Clifton et al., 2020; Quesnel-Vallières et al., 2016; Bernier et al., 2014; Wang et al., 2023).

### Genetic background and molecular basis of syndromic autism

Variations in genetic defects are common in syndromes, with neurodevelopmental, functional psychiatric and behavioral disorders such as ADHD, ASD, and even schizophrenia as clinical manifestations that can vary across individuals and conditions (Morimoto et al., 2021). Many genes with defects impact their encoded proteins and play a role in developing autism, such as the *SHANK3* gene deleted in Phelan-McDermid syndrome, with members of identified gene families contributing to other syndromic autism disorders as well. Furthermore, other gene variants or defects when combined with environmental risk factors may elevate the risk of developing ASD (Hodges et al., 2020), but population frequencies of common syndromic ASD conditions vary considerably. For example, Phenylketonuria involving the *PAH* gene occurs in approximately 1 in 10,000 to 25,000 births (Blau et al., 2010), Prader-Willi syndrome (*SNRPN* and *MAGEL2* genes) in about 1 in 20,000 births (Butler, 2026), Fragile X syndrome (*FMR1* gene) in 1 in 4,000 males and 1 in 8,000 females (Coffee et al., 2009), Phelan-McDermid syndrome (22q13 deletions; *SHANK3* gene) in approximately 1 in 8,000–15,000 individuals (Sarasua et al., 2011), and Tuberous Sclerosis (*TSC1* and *TSC2* genes) in about 1 in 6,000 live births (Jones et al., 2013). These population differences reflect the varying genetic mechanisms underlying each condition, yet all share autism as a common clinical feature, reinforcing the value of studying them together as a model system for understanding ASD pathophysiology from syndromic autism disorders with different genetic defects from single gene variants to deletions involving dozens of genes indicating shared ASD-related pathways.

As the primary focus of this narrative review, ASD is considered within the context of the genetic mechanisms, gene expression patterns, and neurodevelopmental pathways disrupted across the twelve syndromic conditions. The following gene expression data is derived from human sources as documented in OMIM and GeneCards databases unless otherwise specified. To further illustrate, gene expression data from website OMIM and GeneCards sources were used to better understand where and how these genes are expressed across tissues relevant to ASD. As known, all genes are found in all tissues in humans except the red blood cells which do not have a nucleus but expression patterns in tissues can vary from gene to gene. For example, *FMR1* shows notably high expression in the brain and testes, consistent with neurodevelopmental and reproductive features seen in Fragile X syndrome (Genovese A. C. and Butler M. G., 2025; Coffee et al., 2009). *SHANK3* gene is predominantly expressed and concentrated in the brain, particularly in regions governing social behavior and communication such as the prefrontal cortex and hippocampus, consistent with its central role in synaptic scaffolding and the autism phenotype as seen in Phelan-McDermid syndrome, most often from a deletion of the chromosome region harboring this gene (Kolevzon et al., 2014; Zoghbi and Bear, 2012). Also, *UBE3A* demonstrates high expression in the brain with maternally imprinted expression specifically in neurons, explaining why maternal deletion of 15q11-q13 produces profound neurodevelopmental features and autism seen in Angelman syndrome while a paternal deletion produces the distinct phenotype of Prader-Willi syndrome (Godler et al., 2025; Butler, 2026). These tissue-specific expression patterns reinforce why disruption of these genes produces neurodevelopmental consequences and ASD as a primary clinical feature and underscore the importance of understanding gene expression context when considering diagnostic evaluation and potential therapeutic targets for syndromic ASD (Genovese and Butler, 2020; Genovese and Butler, 2024).

Additional factors that contribute to human disease are transposable elements. These elements are found to play a role in neuropathology and autism and are under investigation as these mobile DNA sequences can change position within the genome and disturb function (e.g., 49). These elements are of importance when discussing human genetic disorders particularly related to brain function as they constitute about 50% of the mammalian genome (Techaniyom et al., 2025). They are grouped into two major classes (retrotransposons and DNA transposons) based on their mechanism of action (transposition) affecting genetic function (e.g., 49-53). Retrotransposons, via an intermediate RNA, can expand genomic size which influences gene expression leading to genomic instability with disruption of genes and regulatory regions in chromosomes (Wells and Feschotte, 2020; Bourque et al., 2018; Burns, 2020; Gebrie, 2023). Of interest and as noted, several of our classic genetic disorders with autism do show chromosomal abnormalities including deletions (e.g., Phelan-McDermid, Prader-Willi, Angelman, Williams, DiGeorge).

The most abundant retrotransposons in human DNA are long interspersed nuclear elements (LINEs) such as LINE-1, short interspersed nuclear elements (SINEs) and *Alu* sequences. *Alu* sequences are the most abundant family of repeats in the human genome with more than one million copies which comprise 10% of the genome and implicated in genetic disorders enriched in gene-rich segmental duplications (Price et al., 2004). Hence, insertion of *Alu* repeats in functionally important genes do cause various hereditary disorders, generating the need for further research in neurodevelopmental disorders and autism. Retrotransposition can occur in both germline and embryogenesis including nervous system development with involvement of neural stem cells and neurons. Therefore, an increased number of transposable elements can lead to genetic instability, mosaicism, inheritance and dysregulation of healthy brain function thereby contributing to neurological diseases and mental health including autism (Bodea et al., 2018; Chesnokova et al., 2022).

Transposable elements are primarily regulated by epigenetic mechanisms, specifically DNA methylation with hypomethylation implicated in numerous diseases including brain disorders and cancer (Pappalardo and Barra, 2021). Most transposable elements are transcriptionally inactive due to acquired mutations or epigenetic processes but 8% exert transcriptional activity (Misiak et al., 2019). Maintaining global DNA methylation levels may be critical for both cellular and overall health. However, altered methylation patterns, specifically near neurodevelopmental genes, can impact expression and potentially contribute to neurological and behavioral traits as seen in autism. Several of the genes identified and summarized in our report on syndromic ASD could be impacted. For example, Prader-Willi, Angelman and Fragile X syndromes show alterations in DNA methylation patterns and epigenetics with specific chromosome deletions or abnormalities that may potentially relate to transposable element activity impacting neighboring genes and their expression patterns. This could contribute to abnormal protein production and syndromic-dysmorphic features in addition to altered brain function involving hundreds of recognized genes for autism and their involved mechanisms of action as discussed. This activity should continue to be an area of research for diagnosis and potential treatment of ASD further stimulated by our narrative review.

Preliminary studies of genes found within regions impacted by copy number variation are nominated as driver genes, and their molecular functions, biological pathways, and interactions may overlap in common with ASD (Fernandez and Scherer, 2017; Forrest and Penzes, 2020; Morimoto et al., 2021). This study aimed to identify genes with functional interactive protein mechanisms that overlap and impact pathophysiology with disease causation in classic genetic disorders with ASD, currently lacking attention in the literature. By identifying and characterizing related genes, a designation of syndromes versus non-syndromic ASD may further elevate knowledge necessary to understand the cause and molecular mechanisms or functions for potential treatment of ASD (Fernandez and Scherer, 2017; Forrest and Penzes, 2020; Butler, 2026; Genovese A. and Butler M. G., 2025).

Of the multiple genes associated with ASD (Genovese and Butler, 2020; Butler et al., 2015; Zoghbi and Bear, 2012), some are synapse-related risk genes such as *SHANK3*, while others function as epigenetic-modulating factors such as *MECP2* and *UBE3A* (Sonawane et al., 2021). Others include a broad class of susceptibility genes including *CHD8* and *FMR1*, implicated in transcription and chromatin-remodeling pathways (Remeseiro and Losada, 2013). Rarely have genes been systematically studied for overlap among syndromes with ASD, a focus of this review which is crucial to gain knowledge and to understand or elucidate causation and address treatment options specific to each syndrome, while also appreciating molecular mechanisms that overlap and lead to shared dysfunction across conditions. For example, an illustration using the STRING web-based program and how data were described and summarized included biological processes for SHANK3 are shown in Figure 1.

**FIGURE 1:**
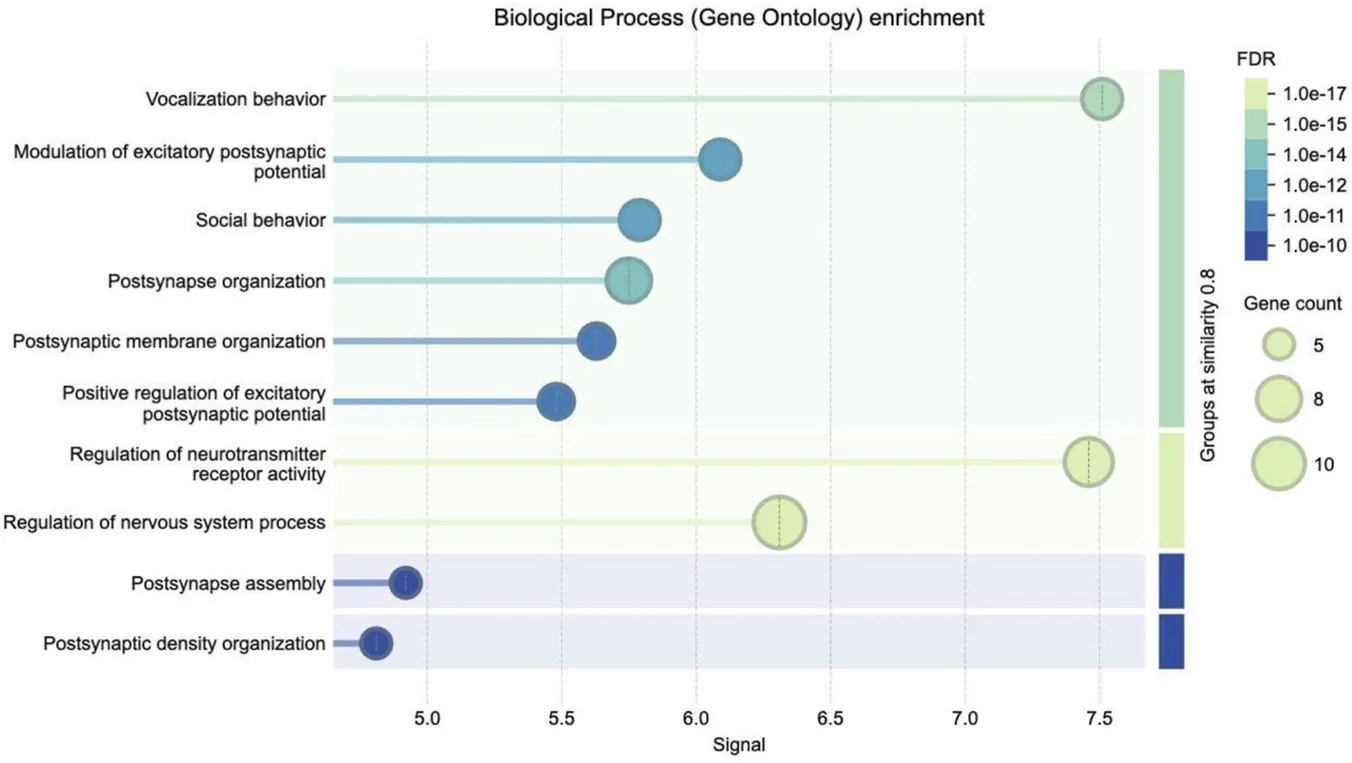
Representative Gene Ontology (Biological Process) enrichment analysis for SHANK3 derived from STRING network analysis. Enriched biological processes identified from the first tier interacting protein network associated with SHANK3 are displayed. Signal strength is shown on the x-axis, bubble size represents the number of genes contributing to each enriched category, and color indicates false discovery rate (FDR)-corrected p-values. This example illustrates the type of functional enrichment output generated for individual causative or implicated genes analyzed across the selected classic syndromic autism spectrum disorders in our study.

Previous studies have established that autism spectrum disorder is genetically heterogeneous and that many classic genetic syndromes including single-gene disorders, chromosomal deletions, and aneuploidies frequently present with ASD features. Prior work has largely focused on identifying individual risk genes, characterizing syndrome-specific phenotypes, or describing molecular pathways within single disorders. However, despite recognition that clinically distinct syndromic conditions share autism as a common clinical outcome, it has remained unclear whether these disorders converge on shared gene networks and molecular pathways at a systems level. This review addresses this gap by integrating ASD-associated genes across multiple classic syndromic autism disorders to identify overlapping genes, protein-protein interaction networks, and convergent biological processes that may underline shared neurodevelopmental phenotypes.

While prior studies have examined individual syndromic autism disorders in isolation, this review provides new insight by systematically integrating ASD-associated genes across multiple clinically distinct syndromes to identify convergence at the network and pathway level. Rather than redefining ASD clinically, this analysis focused on causative and implicated genes from established syndromic conditions with well-documented ASD features, revealing shared molecular processes, biological pathways, and protein interaction networks that may underlie overlapping phenotypes. This systems-level perspective advances existing literature by demonstrating that diverse genetic mechanisms associated with syndromic autism can converge on common molecular dysfunction, offering a unifying framework for understanding shared pathophysiology and informing future pathway-based therapeutic exploration.

### Biological convergence across syndromic ASD conditions

Across the twelve syndromic ASD conditions examined, biological convergence is evident despite the distinct genetic causes underlying each disorder, including chromosomal deletions, single gene variants, and trisomy. Multiple syndromes share enrichment for core neurodevelopmental mechanisms including chromatin remodeling, RNA processing, actin cytoskeleton organization, and synaptic signaling. Recurrent molecular functions include RNA and chromatin binding and serine/threonine kinase activity required for signaling pathways, while shared cellular components predominantly involve the nucleus, chromatin-associated complexes, and the postsynaptic density. Overlapping pathways such as mTOR/AKT signaling, cohesin-related chromatin regulation, and glutamatergic synapse pathways further reinforce this convergence. AKT1-dependent neuroadaptation is associated with the *PTEN* gene, a tumor suppressor that plays an important role in both autism and cancer (Genovese A. and Butler M. G., 2025). Consistent with these mechanistic patterns, shared disease-gene associations frequently include autism, intellectual disability, developmental delay, ADHD or hyperactivity, seizures, anxiety, and behavioral dysregulation. Supporting these mechanistic overlaps, 15 of the 19 overlapping genes are recognized as ASD-associated loci shared across the syndromic ASD conditions, with several ASD-related phenotypes including developmental delay, abnormal social behavior, intellectual disability, repetitive behaviors, anxiety, and broader behavioral dysregulation consistently represented. Together, these findings demonstrate that genetically diverse syndromic ASD converges onto a limited set of molecular systems governing neuronal development and plasticity, synaptic homeostasis, and behavior, which likely underlie their shared overlapping clinical phenotypes.

### Evidence from curated database review: STRING and BioGRID

The 31 studied genes across the twelve syndromic disorders with autism were *SNRPN* and *MAGEL2* for Prader-Willi syndrome, *UBE3A* for Angelman syndrome*, GTF2I, GTF2IRD1, ELN* and *LIMK1* for Williams syndrome*, NIPA1, NIPA2, CYFIP1* and *TUBGCP5* for Burnside-Butler syndrome*, PRODH, COMT, HIRA* and *TBX1* for DiGeorge syndrome, *SHANK3* for Phelan-McDermid syndrome, *PAH* for Phenylketonuria, *MECP2* for Rett syndrome, *FMR1* for Fragile X syndrome, *DYRK1A, SOD1, APP* and *HMGN1* for Down syndrome, *SMC1A, SMC3, NIPBL, RAD21, HDAC8* and *ANKRD11* for Cornelia de Lange syndrome, and *TSC1* and *TSC2* for Tuberous Sclerosis. Thirty of the 31 genes were recognized as ASD-related genes (5–16, 18, 21–25, 27, 28, 30, 31, www.omim.org, www.genecards.org, www.genereviews.org). The expression of these genes is not limited exclusively to the central nervous system. Many are expressed across multiple tissues including the heart, liver, lungs, kidneys, and muscles, as documented in OMIM and GeneCards gene expression data (Godler et al., 2025; Butler, 2026). However, their most clinically relevant expression in the context of ASD is concentrated in brain regions governing social behavior, communication, and cognition, including the prefrontal cortex, hippocampus, amygdala, and basal ganglia (Genovese and Butler, 2020; Zoghbi and Bear, 2012). Disruption of gene function in these regions is thought to underlie the core behavioral features of autism including impaired social communication, restricted and repetitive behaviors, and cognitive dysfunction observed across all twelve syndromic conditions reviewed (Sauer et al., 2021; Genovese and Butler, 2024; Halfon and Kuo, 2013).

To illustrate the nature and significance of gene overlap identified across syndromic ASD, UBE3A and TSC2 served as a representative example of convergence described in Table 2. *UBE3A*, the causative gene in Angelman syndrome, and *TSC2*, one of the causative genes in Tuberous Sclerosis were identified as overlapping genes through STRING first-tier interactive protein network review of shared convergent molecular pathways and mechanisms with direct relevance to ASD pathophysiology. Both genes shared enrichment for mTOR signaling, specifically through the PI3K/AKT/mTOR pathway, which is one of the most well-recognized and important signaling cascades implicated in autism and neurodevelopmental function (e.g., 37). Dysregulation of this pathway disrupts neuronal growth, synaptic plasticity, and protein synthesis in an activity-dependent manner, all of which are processes critical to normal brain development and function. In addition to mTOR pathway overlap, UBE3A and TSC2 share convergence on synaptic signaling pathways, including glutamatergic synapse regulation and postsynaptic density organization, reflecting their combined role in maintaining synaptic protein homeostasis and receptor anchoring at the synapse. At the level of ASD-related human phenotypes, both genes are associated with overlapping clinical features including autism spectrum disorder, intellectual disability, seizures, developmental delay, and behavioral dysregulation, further reinforcing the biological and clinical significance of their molecular convergence (Genovese and Butler, 2020; Genovese and Butler, 2023). This example illustrates how genetically distinct syndromic conditions caused by entirely different genetic defects can converge on shared molecular pathways and produce overlapping neurodevelopmental and behavioral phenotypes, supporting the central premise of this narrative review and highlighting the value of a systems-level approach to understanding syndromic autism.

**TABLE 2:** STRING analysis with convergent molecular pathways and shared genetic mechanisms in classic syndromic autism spectrum disorders*.

Syndrome (gene) [overlapping Gene(s)]	Syndromic ASD connection [overlapping Gene(s)]	Biological processes	Molecular functions	Cellular components	KEGG pathways/Reactome pathways	Disease-gene associations	ASD-related human phenotypes
+Prader-Wili syndrome (SNRPN, MAGEL2) [UBE3A]	Prader-Willi syndrome (MAGEL2) Angelman syndrome [UBE3A]	mRNA splicing; RNA processing	snRNA binding	snRNP complex	Spliceosome/RNA transport/mRNA splicing	​	Impaired social behavior; developmental delay; repetitive behaviors
+Angelman syndrome (UBE3A) [DLG1, TSC2, RAD23A]	Angelman syndrome [DLG1, RAD23A] Prader-Willi syndrome [MAGEL2] Tuberous Sclerosis [TSC2]	Protein ubiquitination	Ubiquitin ligase activity	Ubiquitin ligase complex	Ubiquitin-mediated proteolysis; protein degradation/Synaptic signaling	Colon carcinoma; Nephroblastoma; Colorectal carcinoma	Non-verbal; Impairment in personality functioning; intellectual disability; ataxia
+Burnside-Butler syndrome (NIPA1) [CYFIP1, NIPA2, TUBGCP5]	Burnside-Butler syndrome (CYFIP1, NIPA2, TUBGCP5)	Axonogenesis	​	Endoplasmic reticulum tubular network	​	Hereditary spastic paraplegia; hereditary spastic paraplegia 2, 10, 13, 30; chromosome 15q11.2 deletion syndrome; Neurodegenerative disease; Dyscalculia; hereditary spastic paraplegia 3A; Troyer syndrome; MASA syndrome; autosomal dominant disease; autosomal genetic disease; nervous system disease	Neurodevelopmental delay; language and motor delays; attention deficit
(NIPA2) [CYFIP1, NIPA1, TUBGCP5]	Burnside-Butler syndrome (CYFIP1, NIPA1, TUBGCP5)	​	Magnesium ion transmembrane transporter activity	​	Transport of small molecules/Miscellaneous transport and binding events	Chromosome 15q11.2 deletion syndrome; Dyscalculia; atypical hemolytic-uremic syndrome; Hemolytic-uremic syndrome; autosomal dominant disease; Monogenic disease	Neurodevelopmental delay
(CYFIP1) [FMR1, FXR1, FXR2]	Fragile X syndrome [FMR1, FXR1, FXR2]	Positive regulation of Arp2/3 complex mediated actin nucleation	Translation regulator activity	SCAR complex; Filopodium; Filopodium tip	Regulation of actin cytoskeleton/Bacterial invasion of epithelial cells	Fragile X syndrome	
(TUBGCP5) [NIPA1, NIPA2, CYFIP1]	Burnside-Butler syndrome (NIPA1, NIPA2, CYFIP1)	Microtubule nucleation	Gamma tubulin binding	Gamma tubulin small and large complexes	Recruitment of mitotic centrosome proteins and complexes/Miscellaneous transport and binding events	Chromosome 15q11.2 deletion syndrome; Dyscalculia; Congenital nervous system abnormality	Abnormality of coordination; Dysarthria; Distal sensory impairment; Involuntary movements; functional motor deficit; Dyscalculia; Obsessive-compulsive disorder
+Phelan- McDermid syndrome^1^ (SHANK3) [DLG4]	Phelan-McDermid syndrome [DLG4]	Regulation of neurotransmitter receptor activity	Scaffold protein binding	Excitatory/glutamatergic synapse	Glutamatergic synapse/Neurexins and neuroligins	Autistic disorder; Asperger syndrome; developmental disorder of mental health; Gilles de la Tourette syndrome	Social impairment; repetitive behaviors; developmental delay; abnormal social-emotional behavior
++Phenylketonuria (PAH) [DDC]	Phenylketonuria [DDC] DiGeorge syndrome [COMT]	Axon guidance	Netrin receptor activity	Plasma membrane	Axon guidance/Neural development	Congenital mirror movement disorder; Agenesis of the corpus callosum	Motor coordination deficits; abnormal brain connectivity; atypical lateralization and mild cognitive impairments
**Fragile X syndrome (FMR1) [FXR1, FXR2, CYFIP1]	Fragile X syndrome [FXR1, FXR2] Burnside-Butler syndrome [CYFIP1]	Negative regulation of translation	RNA binding, translation repressor	RISC complex	RNA transport; miRNA regulation/Synaptic regulation	Fragile X syndrome; Fragile X-associated tremor/ataxia syndrome; X- linked monogenic disease	Autism; intellectual disorders; ADHD
+++Down syndrome (DYRK1A) [RAD54L2]	Down syndrome [RAD54L2]	​	​	​	​	​	Autism; intellectual disorders
**Cornelia de Lange syndrome^2^ (SMC1A) [RAD21, SMC3]	Cornelia de Lange syndrome (RAD21, SMC3)	Sister chromatid cohesion	Chromatin binding	Cohesin complex	Cell cycle/Cohesin loading onto chromatin	Cornelia de Lange syndrome 2; Cornelia de Lange syndrome 3; Roberts syndrome	Autism; intellectual disability; ADHD; OCD-like behaviors; seizures
(SMC3) [SMC1A, RAD21]	Cornelia de Lange syndrome (SMC1A, RAD21)						
(RAD21) [SMC1A, SMC3]	Cornelia de Lange syndrome (SMC1A, SMC3)	Separation of sister chromatids	Chromosomal segregation machinery	Chromosomal arm and centromere	Cohesin loading and separation	Cornelia de Lange syndrome 4	Neurodevelopmental delay; autism spectrum disorder; anxiety; seizures
**Tuberous Sclerosis (TSC1) [AKT1, TSC2] (TSC2) [AKT1, UBE3A]	Tuberous Sclerosis [AKT1], (TSC2)	mTOR signaling	GTPase binding	TSC1-TSC2 complex	mTOR signaling pathway/AKT-mTOR signaling	Lymphangioleiom yomatosis; kidney angiomyolipoma; Subependymal giant cell astrocytoma; Angiomyolipoma, lung disease; Fibroma	Autistic and aggressive behaviors; autism; Self- injurious behaviors, abnormal social behavior; anxiety; Depression; abnormal emotion/affect behavior; IDD; hyperactivity; specific learning disability; Precocious puberty; behavioral abnormality
	Tuberous Sclerosis [AKT1] Angelman syndrome [UBE3A]	Cell growth; survival	Serine/threonine kinase activity	Cytoplasm	mTOR signaling/PI3K-Akt signaling	*Proteus* syndrome; Overgrowth disorder	Behavioral abnormalities; intellectual disability; somatic mosaicism

* STRING, program and database: + Deletion disorder, ++ Single gene disorder, ** More than one candidate gene disorder, and +++ Trisomy.

No information available is represented by gray color region in the columns.

Using STRING, web-based program with criteria as described in the MATERIALS, and METHODS, section for Count in Network, Signal, Strength and FDR, to establish significance per finding in each category (e.g., biological processes, Molecular Functions, etc.) with p-values generated (at least p < 0.05) for each finding with most significant feature located at the top of each category. Genes bolded in red represent overlapping causative or implicated genes identified across multiple syndromic autism disorders, reflecting shared genetic architecture. Notably, 15 of these 19 overlapping genes are established ASD-associated genes. Entries bolded in red indicate KEGG/Reactome pathways and ASD-related human phenotypes that are shared across multiple implicated genes.

DLG4 encodes PSD-95, a major post synaptic scaffolding protein essential for synaptic plasticity and excitatory signaling. Disruptions in DLG4 have been associated with neurodevelopmental phenotypes overlapping those observed in Angelman syndrome and Tuberous Sclerosis. together, these connections highlight DLG4 as a shared molecular node in the pathogenesis of multiple syndromic autism disorders.

RAD21 is part of the broader RAD, superfamily involved in SNA, repair and chromosomal stability. Although functionally distinct, RAD21, RAD23A, and RAD54L2 all contribute to maintaining genomic integrity. These genes reflect the molecular convergence of chromatin regulation and neurodevelopment, a recurrent them across syndromic autism-associated loci.

The STRING database evidence identified 19 genes (i.e., *SNRPN, UBE3A, DLG1, TSC2, RAD23A, CYFIP1, NIPA2, TUBGCP5, NIPA1, FMR1, FXR1, FXR2, DDC, DLG4, RAD54L2, RAD21, SMC3, SMC1A,* and *AKT1*) showing overlap and contribution to autism with shared molecular functions. Fifteen of these genes are recognized in the existing literature as ASD-related genes. For example, Prader-Willi syndrome and Tuberous Sclerosis share overlapping first-tier interactive genes with Angelman syndrome, known for its *UBE3A* gene defect. In addition, genes involved with Burnside-Butler syndrome are components of the typical 15q11.2-q13 deletion common in both Prader-Willi and Angelman syndromes which includes the proximal 15q11.2 BP1-BP2 region (see Table 2). About one fourth of all patients with Prader-Willi and Angelman syndromes have this region deleted (Butler and Duis, 2020; Rafi and Butler, 2020; Godler et al., 2025; Butler, 2023). The STRING data shows that interactive *FXR1* and *FXR2* genes overlap with both Burnside-Butler and Fragile X syndromes, along with CYFIP1 which plays an important role in both genetic disorders. Another notable example is DDC, which shows overlap in the first-tier protein-protein dataset as illustrated in Table 2 and shared with Phenylketonuria and DiGeorge syndrome with the gene found on chromosome 7. It provides instructions for encoding the aromatic L-amino acid decarboxylase enzyme, vital for brain and nervous system function through two neurotransmitters, dopamine and serotonin, with spliced transcript variants encoding different protein isoforms (e.g., 48).

The STRING analysis protein network of the first-tier or ten associated or interactive proteins and their overlapping, causative or implicated genes from the twelve classic syndromic autism disorders are shown in Table 2. Hence, the tabular components are formatted showing the causative and implicated syndromic genes, their highlighted overlapping genes across the selected syndromes, and convergent molecular pathways with mechanisms including biological processes, molecular functions, cellular components, KEGG and Reactome pathways, disease-gene associations and ASD-related human phenotypes. Furthermore, overlapping genes across two or more syndromic conditions are highlighted in red within Table 2, allowing for visual identification of convergent genetic contributors across clinically distinct disorders. As shown in Table 2, the overlapping genes share enrichment for core neurodevelopmental mechanisms including chromatin remodeling, RNA processing, synaptic signaling, and protein homeostasis despite arising from diverse underlying genetic defects. Recurrent molecular functions identified across multiple syndromes include RNA and chromatin binding, serine/threonine kinase activity, and ubiquitin ligase activity, while shared cellular components predominantly involve the nucleus, chromatin-associated complexes, and the post synaptic density. Shared KEGG and Reactome pathway annotations further reinforce convergence across syndromes, with mTOR signaling, glutamatergic synapse pathways, and cohesin-related chromatin regulation among the most recurrently represented. Consistent with these mechanistic overlaps, shared disease-gene associations and ASD-related human phenotypes are captured in Table 2 that frequently include autism, intellectual disability, developmental delay, seizures, anxiety, repetitive behaviors, and abnormal social behavior, underscoring the biological relevance of these overlapping genes to the shared neurodevelopmental and behavioral phenotypes observed across the twelve syndromic ASD conditions.

The existing BioGRID data on biological processes, molecular functions, and cellular components further highlights the collective role of the representative genes in autism pathophysiology. For example, LIMK1 contributes to axon guidance, nervous system development, and axon extension consistent with its localization to neuron projections and functions as a serine/threonine protein kinase. SOD1 is found to be associated with Amyotrophic Lateral Sclerosis with a canonical antioxidant role and participation in axon transport, neurofilament organization, neuronal survival, and myelin maintenance. It functions in Rac GTPase and calcineurin binding, and superoxide dismutase activity localized to dendrites and neuronal cell bodies supporting a role in autism. *SOD1* is also one of the nine genes found on chromosome 21 recognized as an autism-related gene in Down syndrome. In addition, TSC1 regulates cytoskeletal remodeling through Rho GTPase signaling and stress fiber assembly, underscoring its role in actin-dependent neuronal processes.

The SHANK3 encoded protein is enriched for processes such as dendritic spine morphogenesis, synapse assembly, postsynaptic density organization, learning, memory, and glutamatergic transmission, reflecting its binding to long Homer scaffold proteins (e.g., Homer 1b/c), GKAP, and ionotropic glutamate receptors within the postsynaptic density; all contributing to autism. Finally, UBE3A is directly linked to brain development and circadian rhythm regulation through its ubiquitin-protein transferase activity, consistent with its central role in synaptic protein turnover and autism. Together, data from these representative genes found in our review emphasize the convergence of autism-related syndromes on pathways governing neuronal connectivity, synaptic plasticity, and protein homeostasis, reinforcing their biological relevance to ASD.

### Chromatin remodeling and epigenetic regulation

Despite clinical and genetic heterogeneity, syndromic autism disorders such as Rett, Angelman, Fragile X, Phelan-McDermid, and Cornelia de Lange syndromes share notable similarities in core molecular pathways. A particularly recurrent theme across multiple syndromes is disruption of chromatin remodeling and epigenetic regulation, important for future research on therapeutic interventions. A notable example is the cohesin complex, where *NIPBL, RAD21, SMC1A, SMC3,* and possibly *RAD23A, RAD54L2,* and *TUBGCP5* genes and their encoded proteins may potentially contribute to sister chromatid cohesion, cell cycle regulation, homologue activity, and chromatin architecture (Remeseiro and Losada, 2013). Disruption of this complex could play a role in Cornelia de Lange syndrome and affect genome topology with long-range enhancer-promoter interactions, broadly impairing gene expression programs critical for neurodevelopment (Kline et al., 2018). Additionally, RAD21 in Cornelia de Lange syndrome, RAD23A in Angelman syndrome, and RAD54L2 in Down syndrome share similar functions including chromatin remodeling, homologous recombination, nucleotide excision repair, and cohesin complex chromatin separation, which relates to the genetic causation and clinical findings associated with these disorders.

Epigenetic regulation via histone deacetylation emerges as another recurrent theme as previously discussed with transposable elements and roles, with *MECP2, GTF2I*, and *HDAC8* genes participating in histone deacetylase complexes that mediate transcriptional repression. The *HDAC8* and *ANKRD11* genes noted in Cornelia de Lange syndrome also play a role in chromatin and histone function, contributing to gene regulation. *ANKRD11* is also a recognized autism gene found in the 16q24.3 region and when deleted or mutated causes KBG syndrome (Youngs et al., 2011), characterized by neurological deficits with localization of the protein to the nuclei of neurons playing a role in neural plasticity including intellectual disabilities along with short stature, characteristic triangular facies, macrodontia, and skeletal abnormalities (Sirmaci et al., 2011; Gao et al., 2025). The HDAC8 component is particularly relevant in disorders like Rett and Williams syndromes, where dysregulated chromatin remodeling alters neuronal plasticity and synaptic function (Lyst and Bird, 2015). Beyond previously discussed chromatin regulators in the cohesin complex, distinct transcriptional regulators such as HIRA and ANKRD11 in DiGeorge and Cornelia de Lange syndromes point to parallel chromatin remodeling pathways that impact early gene expression programs, and both have been increasingly recognized as contributors to neurodevelopmental disorder phenotypes through their effects on chromatin accessibility and transcriptional dynamics (Hutsler and Zjang, 2010; Estes and McAllister, 2015). Given the reversibility of epigenetic modifications, these genes may represent particularly attractive therapeutic targets for future investigation.

TUBGCP5 plays a role in mitosis and could contribute to chromosome non-disjunction noted in uniparental disomy 15 in both Prader-Willi and Angelman syndromes and trisomy 21 in Down syndrome (Rafi and Butler, 2020). The *UBE3A* gene is imprinted and encodes an E3 ubiquitin-protein ligase that is part of the ubiquitin protein degradation system and causes Angelman syndrome when disturbed. *UBE3A* is maternally expressed in the brain, while the maternal and paternal alleles are expressed in other tissues such as the heart, lungs, liver, and muscles, emphasizing its role in neurodevelopment specifically (Godler et al., 2025). The 15q11-q13 deletion of maternal origin causes Angelman syndrome, characterized by motor and intellectual disability, poor coordination, ataxia, hypotonia, epilepsy, absence of speech, and unique facies with bouts of laughter.

### RNA regulation and the spliceosome network

The spliceosome and RNA regulation network, highlighted by SNRPN and FMR1, implicates defects in mRNA splicing, translation repression, and processing that are essential to the development and function of neurons with high transcriptional complexity. These RNA-binding proteins and complexes such as snRNPs and RISC are foundational to post-transcriptional regulation, a vulnerability seen in both Prader-Willi and Fragile X syndromes (Zoghbi and Bear, 2012). Impaired RNA metabolism is increasingly recognized as a common mechanism underlying intellectual disability and autism (Toma et al., 2014). *FXR1* and *FXR2* genes encode RNA-binding proteins containing 2 KH domains and one RGG box. They interact functionally with similar proteins associated with FMR1 seen in Fragile X syndrome and with CYFIP1, which plays a combined role in both Burnside-Butler and Fragile X syndromes. These proteins shuttle between the nucleus and cytoplasm and are associated with polyribosomes, predominantly with the 60S ribosomal subunit and protein production. FXR2 also plays a role in cognitive disability, a common finding in Fragile X syndrome. The convergence of RNA regulatory dysfunction across multiple syndromic ASD conditions underscores the critical importance of post-transcriptional gene regulation in normal neurodevelopment and the vulnerability of neuronal function to disruptions in this system.

### Synaptic signaling and protein homeostasis

Other shared molecular patterns include the ubiquitin-proteasome system, where the *UBE3A* gene is disturbed in Angelman syndrome and FMR1 gene disturbance in Fragile X syndrome may play key roles in proteostasis and synaptic protein turnover, as well as synaptic scaffolding and signaling, with SHANK3, UBE3A, and FMR1 regulating glutamatergic synaptic activity. Dysfunction across these axes can impair synaptic plasticity, a hallmark of ASD pathophysiology (Zoghbi and Bear, 2012). Disruptions in genes regulating synaptic connectivity and neurotransmitter receptor signaling have direct consequences for cortical circuits involved in speech and language processing, particularly within the prefrontal and temporal cortices. Altered dendritic spine maturation and cadherin-mediated adhesion can destabilize neuronal networks, while impaired growth factor signaling further compromises synaptic plasticity and long-range connectivity across regions such as the hippocampus and amygdala. Together these convergent pathways illustrate how diverse genetic lesions in syndromic autism manifest as shared disturbances in neural systems underlying communication and social behavior (Estes and McAllister, 2015). Convergence at the synapse through dysregulated protein synthesis, degradation, and receptor anchoring may represent a final common pathway across many neurodevelopmental syndromes. Functionally, this overlap is supported by shared enrichment in pathways such as cohesin loading onto chromatin, HDAC-mediated histone deacetylation, mTOR signaling, and RNA transport, all of which are implicated in neural development and behavior. Importantly, genes such as *SHANK3, FMR1, ANKRD11, MECP2, MAGEL2, CYFIP1, TUBGCP5,* and *UBE3A* are involved in pathways and proteins regulating social behavior, language acquisition, and cognitive function, reinforcing their centrality in ASD phenotypes common to the syndromic autism disorders studied in this review. These patterns of shared dysfunction are found to be particularly prominent in Phelan-McDermid, Fragile X, Rett, Prader-Willi, Cornelia de Lange, Burnside-Butler, and Angelman syndromes.

### Emerging and underexplored convergent pathways

Extending analysis to higher-confidence tier-level ASD genes reveals further functional convergence across syndromic ASD conditions, notably, calcium signaling emerged as a shared pathway among GTF2IRD1, GTF2I, and SHANK3 which contributes to calcium-activated potassium channel activity or calcium-regulated synaptic modulation. This implicates altered neuronal excitability and calcium-dependent transcription in ASD pathogenesis, particularly within Williams and Phelan-McDermid syndromes (Sztainberg et al., 2016). Furthermore, underexplored pathways such as the complement and coagulation cascade highlighted by *NIPA2*, a cation/magnesium transporter gene (Rafi and Butler, 2020), could play a role in immune-neural interactions in select ASD subtypes, possibly linking systemic inflammation or complement dysregulation with atypical neurodevelopment (Estes and McAllister, 2015). Additional patterns highlight the role of endosomal trafficking and mitochondrial regulation in ASD risk. Genes such as *APP, MAGEL2,* and *SHANK3*, which play a role in Down, Prader-Willi, and Phelan-McDermid syndromes, display convergence on early endosome-related pathways, indicating potential dysfunction in receptor recycling and synaptic protein turnover, key processes in neuronal pruning and synaptic plasticity (Genovese A. and Butler M. G., 2025; Zoghbi and Bear, 2012). Similarly, PRODH and SOD1, which play a role in DiGeorge and Down syndromes and are implicated in mitochondrial pathways and oxidative stress management, underscore the role of redox imbalance and energy metabolism in ASD-related neurobiology (Giulivi et al., 2010). These emerging pathways, while less studied than chromatin remodeling or synaptic signaling, represent important areas for future investigation and may help explain aspects of ASD pathophysiology not fully accounted for by currently recognized mechanisms.

## Conclusions, therapeutic implications and future directions

The observations based on searching existing web-based data from the literature and other sources underscored the importance of conceptualizing syndromic autism not as genetically disparate disorders but as conditions that converge on a set of interrelated core neurodevelopmental pathways. Recognizing this shared biology may enhance diagnostic precision and promote therapeutic repurposing across multiple but related syndromes with different clinical presentations. Potential therapeutic strategies suggested by these convergent pathways include small molecule inhibitors targeting HDACs or mTOR signaling, or agents that modulate RNA splicing or synaptic protein degradation, which may have utility across distinct ASD subtypes. Based on the breadth of its representation across multiple syndromic conditions, the mTOR/AKT signaling pathway emerges as the most recurrently disrupted and broadly impactful pathway identified in this review. This pathway was implicated across Tuberous Sclerosis, Angelman, Fragile X, Phelan-McDermid, and Down syndrome through genes including TSC1, TSC2, UBE3A, FMR1, SHANK3, and AKT1, suggesting it represents a primary convergent vulnerability across syndromic ASD conditions and a particularly promising target for pathway based therapeutic intervention. Regarding core ASD phenotypes, genes involved in synaptic signaling and glutamatergic transmission including SHANK3, FMR1, and UBE3A are most strongly associated with social communication deficits and are most enriched in Phelan-McDermid, Fragile X, and Angelman syndromes. Genes involved in chromatin remodeling and RNA regulation including MECP2, SNRPN, and HDAC8 are more broadly associated with repetitive behaviors and cognitive rigidity and are most enriched in Rett, Prader-Willi, and Cornelia de Lange syndromes. Several of these associations are supported by preclinical animal model studies, and a comprehensive phenotype specific gene mapping analysis represents an important direction for future investigation.

However, several limitations should be considered such as our review that did not include laboratory experiments to test the shared biological findings or undertake clinical trials. Non-specific environmental influences and epigenetic modifications beyond the scope of the selected syndromic gene defects were not examined in this review, as our focus was specifically on causative and implicated genetic factors within established syndromic ASD conditions related to different genetic defects. While bioinformatics enrichment and database synthesis can identify patterns of functional similarity, they do not directly establish causality or temporal sequence. Additionally, the functional annotation of some genes is often incomplete or unclear, especially for non-coding RNAs and low-expression transcripts. Experimental validation in relevant neuronal models including patient-derived iPSCs, single-cell RNA sequencing of brain tissue, and organoids will be essential to determine the actual contribution of these genes to phenotypes. Additionally, emerging evidence suggests that transposable genetic elements and non-coding DNA and RNA molecules may play important roles in ASD onset and progression, as well as in human fetal brain development. Future studies comparing the convergent syndromic pathways identified in this review against disrupted signaling and gene expression patterns in idiopathic ASD would be a valuable and important next step in establishing the broader relevance of these findings beyond classic syndromic conditions. Several genes examined in this review including RAD54L2 have incomplete functional annotation in current databases, reflecting the current state of published literature for these less characterized genes rather than an omission in our analysis. Conclusions about their overlapping function were drawn from available STRING first tier network interactions and existing published literature, and future experimental studies will be essential to fully characterize these genes and validate the network interactions identified here. Non-specific environmental influences and a comprehensive comparison of global and population specific prevalence data across all twelve syndromic conditions also represent areas beyond the scope of this review that warrant further investigation. While beyond the scope of this narrative review, future studies examining the contribution of these elements to syndromic ASD pathophysiology would be a valuable complement to the gene-protein network approach.

Together, the findings synthesized in this review suggest that diverse syndromic ASD genes disrupt a set of core cellular systems including chromatin remodeling, RNA regulation, processing and inhibition, protein isoform development, proteostasis, and synaptic signaling and control. These mechanistic similarities may help explain the overlapping clinical phenotypes observed across distinct syndromes such as intellectual disability, language delay, and autism spectrum behaviors. By framing syndromic ASD as a spectrum of disruptions to interconnected molecular systems, a foundation is established for unified therapeutic strategies that transcend traditional gene-by-gene approaches. These shared pathways offer promising therapeutic entry points, as future interventions may target dysregulated mTOR or HDAC activity, modulate RNA splicing and translation machinery, or enhance synaptic protein development, processing, and turnover. This offers a strong framework for pathway-based, in addition to gene-specific, treatment strategies (Sztainberg et al., 2016).

In summary, this narrative review represents an effort to identify shared ASD-related genes in selected classic syndromic ASD conditions and demonstrates a novel observation that 30 of their 31 causative or implicated genes are recognized as playing a role in autism. Nineteen genes showed an overlap with shared molecular mechanisms, processes, and pathways through study of existing computational and curated database evidence as illustrated in Figure 2. Diverse genetic etiologies associated with autism converge on shared molecular pathways and protein-protein interactive networks, with core convergence observed in chromatin remodeling, RNA processing, and synaptic signaling, reinforcing that syndromic ASD arises from disruption of a limited number of critical neurodevelopmental structural or functional systems. The search of higher-tier database findings revealed converging themes including calcium signaling via potassium channel regulation, complement cascade involvement suggesting neuroimmune dysfunction, and shared roles in early endosome and mitochondrial pathways.

**FIGURE 2:**
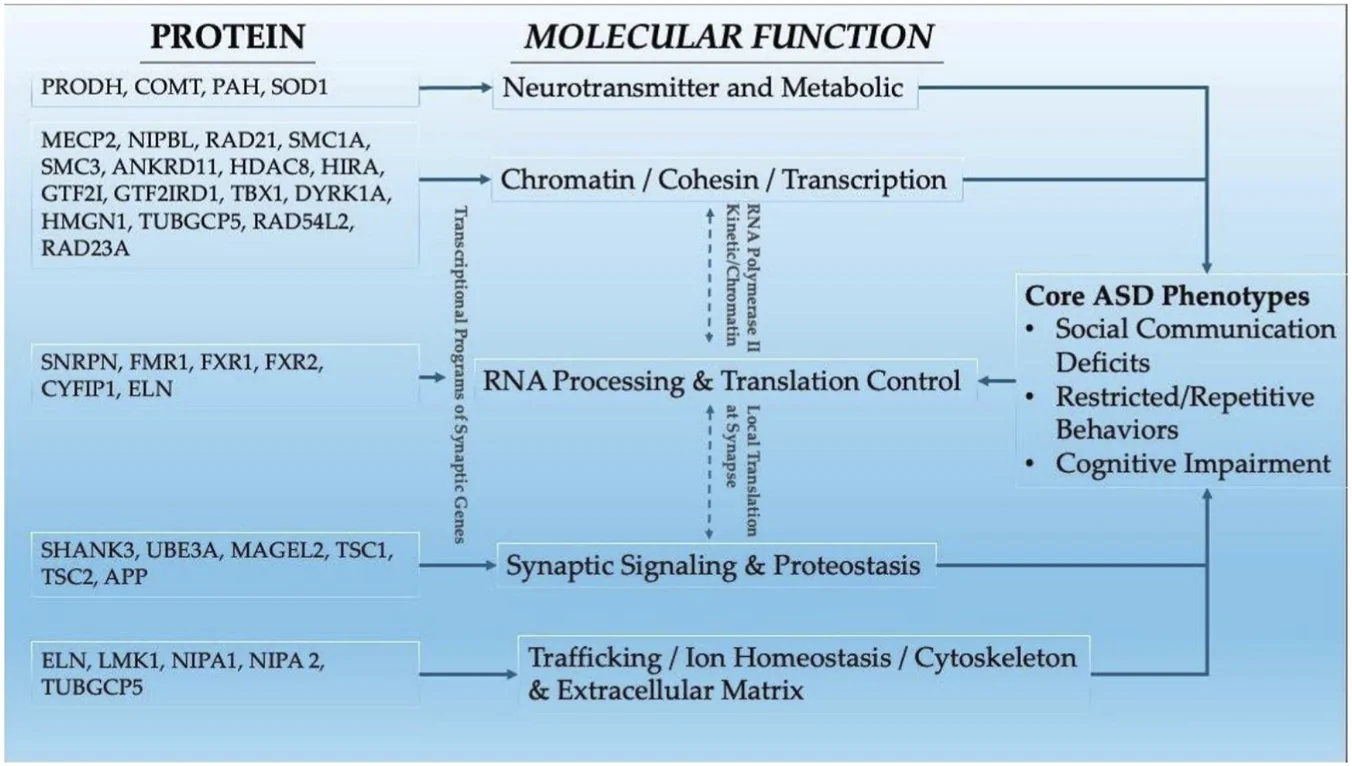
Functional modules and convergent pathways in syndromic autism spectrum disorders (ASD). Solid arrows indicate convergence of modules onto core ASD phenotypes (social communication deficits, restricted/repetitive behaviors, cognitive impairment), while dashed arrows represent cross-talk between modules. Key interactions include Chromatin/Cohesin → RNA Processing (co-transcriptional splicing), Chromatin → Synaptic (transcriptional control of synaptic genes), and RNA Processing → Synaptic (activity-dependent local translation). Modules denote primary functional roles, though several genes act in multiple processes. Shown are causative or interactive genes across 12 syndromic ASD conditions and along with shared genes amongst syndromes; for example, TBX1 and DYRK1A are categorized in the Chromatin/Cohesin/Transcription module for their roles in transcriptional regulation and neurodevelopment, though both may indirectly affect synaptic phenotypes. Other representative examples are noted in the literature (44- 47).

Our review supports a systems-level framework for understanding syndromic autism, emphasizing that shared neurodevelopmental phenotypes may arise from disruption of common molecular processes rather than isolated genetic mechanisms, and providing a necessary foundation for future studies aimed at pathway-based stratification and potential therapeutic development in autism spectrum disorders. These findings support a model for ASD pathophysiology in which distinct genetic variants may disrupt interconnected cellular systems directly through syndromic ASD genes or by influencing activity and function of other interactive or related genes and their encoded proteins. Alterations could lead to ASD, individually or through intergenic interactions and mechanisms. Understanding convergent networks and shared connections would strengthen the case for pathway-targeted interventions that may apply across genetically heterogenous syndromes with growing evidence of shared interactive functions and causative disease mechanisms. As our understanding increases, future research using functional validation in experimental neurological tissue models will shift these insights into personalized therapeutic strategies for individuals affected with syndromic forms of autism and relationship to ASD alone.
